# Counteraction of Biofilm Formation and Antimicrobial Potential of *Terminalia catappa* Functionalized Silver Nanoparticles against *Candida albicans* and Multidrug-Resistant Gram-Negative and Gram-Positive Bacteria

**DOI:** 10.3390/antibiotics10060725

**Published:** 2021-06-16

**Authors:** Mohammad Azam Ansari, Abul Kalam, Abdullah G. Al-Sehemi, Mohammad N. Alomary, Sami AlYahya, Mohammad Kashif Aziz, Shekhar Srivastava, Saad Alghamdi, Sultan Akhtar, Hussain D. Almalki, Syed F. Adil, Mujeeb Khan, Mohammad R. Hatshan

**Affiliations:** 1 Department of Epidemic Disease Research, Institute for Research & Medical Consultations (IRMC), Imam Abdulrahman Bin Faisal University, Dammam 31441, Saudi Arabia; maansari@iau.edu.sa; 2 Department of Chemistry, Faculty of Science, King Khalid University, Abha 61413, Saudi Arabia; agsehemi@kku.edu.sa; 3 Research Center for Advanced Materials Science (RCAMS), King Khalid University, Abha 61413, Saudi Arabia; 4 National Centre for Biotechnology, King Abdulaziz City for Science and Technology (KACST), Riyadh 11442, Saudi Arabia; malomary@kacst.edu.sa (M.N.A.); salyahya@kacst.edu.sa (S.A.); 5 Department of Chemistry, Faculty of Science, University of Allahabad, Allahabad 211001, Uttar Pradesh, India; mohdkashifaziz@allduni.ac.in (M.K.A.); diriids@allduni.ac.in (S.S.); 6 Laboratory Medicine Department, Faculty of Applied Medical Sciences, Umm Al-Qura University, Makkah 24231, Saudi Arabia; ssalghamdi@uqu.edu.sa; 7 Department of Biophysics, Institute for Research & Medical Consultation (IRMC), Imam Abdulrahman Bin Faisal University, Dammam 31441, Saudi Arabia; suakhtar@iau.edu.sa; 8 Department of Chemistry, University College in Al-Qunfudah, Umm Al-Qura University, Makkah Al-Mukarramah 1109, Saudi Arabia; hdmalki@uqu.edu.sa; 9 Department of Chemistry, College of Science, King Saud University, Riyadh 11451, Saudi Arabia; kmujeeb@ksu.edu.sa (M.K.); mhatshan@ksu.edu.sa (M.R.H.)

**Keywords:** biofilm, antibiotics resistant, bio-actives capping, silver nanoparticles, TEM, ultrastructural changes, *Candida albicans*, MRSA

## Abstract

Biofilms not only protect bacteria and Candida species from antibiotics, but they also promote the emergence of drug-resistant strains, making eradication more challenging. As a result, novel antimicrobial agents to counteract biofilm formation are desperately needed. In this study, *Terminalia catappa* leaf extract (TCE) was used to optimize the TCE-capped silver nanoparticles (TCE-AgNPs) via a one-pot single-step method. Varied concentrations of TCE have yielded different sized AgNPs. The physico-chemical characterization of TCE-AgNPs using UV-Vis, SEM, TEM, FTIR, and Raman spectroscopy have confirmed the formation of nanostructures, their shape and size and plausible role of TCE bio-active compounds, most likely involved in the synthesis as well as stabilization of NPs, respectively. TCE-AgNPs have been tested for antibiofilm and antimicrobial activity against multidrug-resistant *Pseudomonas aeruginosa* (MDR-PA), methicillin-resistant *Staphylococcus aureus* (MRSA), and *Candida albicans* using various microbiological protocols. TCE-Ag-NPs−3 significantly inhibits biofilm formation of MDR-PA, MRSA, and *C. albicans* by 73.7, 69.56, and 63.63%, respectively, at a concentration of 7.8 µg/mL, as determined by crystal violet microtiter assay. Furthermore, SEM micrograph shows that TCE-AgNPs significantly inhibit the colonization and adherence of biofilm forming cells; individual cells with loss of cell wall and membrane integrity were also observed, suggesting that the biofilm architecture and EPS matrix were severely damaged. Moreover, TEM and SEM images showed that TCE-AgNPs brutally damaged the cell wall and membranes of MDR-PA, MRSA, and *C. albicans*. Additionally, extreme ultrastructural changes such as deformation, disintegration, and separation of cell wall and membrane from the cells, have also been observed, indicating significant loss of membrane and cell wall integrity, which eventually led to cell death. Overall, the research revealed a simple, environmentally friendly, and low-cost method for producing colloidal TCE-AgNPs with promising applications in advanced clinical settings against broad-spectrum biofilm-forming antibiotic-resistant bacteria and candida strains.

## 1. Introduction

The Gram-negative multidrug-resistant *Pseudomonas aeruginosa* (MDR-PA) and Gram-positive methicillin-resistant *Staphylococcus aureus* (MRSA) human pathogens are the leading causes of nosocomial infections in hospitals globally [1,2]. In the same line, colonization of *Candida albicans* in urinary catheters and endotracheal tubes implanted in immunocompromised patients indicates the alarming frequency of fungal infections [3,4]. Matured biofilms of MDR-PA, MRSA, and *C. albicans* are comprised of a complex three-dimensional structure of multiple stacked layers and aggregated clusters of their cells, hyphae, extracellular DNA, proteins, and abundant exopolysaccharide (EPS) matrix [5,6,7]. Establishment and maturation of biofilms of such microbial pathogens on a number of medical devices such as dental restorative fillings, orthopedic implants, contact lenses, and catheters have been accounted majorly for the failure of biomaterial-based medical implants and traditional antibiotics. Thus, the menace of antimicrobial resistance (AMR) in biofilms has fueled the hospital acquired infections further. To control microbial biofilms, a variety of medicinal crude extracts and essential oils containing bio-active carbohydrates, terpenoids, polyphenols, alkaloids, phenolic acids, and proteins have been used as well as these bio-actives-capped metal nanoparticles have been employed such as potential antimicrobial, antibiofilm, and antiquorum sensing agents [8,9].

Nanoparticles (NPs) are referred to as structures that possess at least one of its dimensions less than 100 nm. Such nanoscale fabrication of organic, inorganic, metallic, and biological structures is generally done by following either bottom-up or top-down approaches. In modern times, green approaches for NPs synthesis have gained immense interest due to its eco-friendly processing and low-cost input [10,11,12]. Furthermore, NPs derived from various plant extracts contain a variety of indigenous bio-active molecules such as polyphenols, alkaloids, terpenoids, sugars, proteins, and enzymes that act as reducing, capping, and stabilizing agents. Among the various plants reported for the fabrication of NPs is the *Terminalia catappa* plant, which has been popularly known as a folk medicine due to its promising therapeutic effects against dermatitis and hepatitis in India and the Philippines [13]. It has been found that *Terminalia catappa* leaf extract (TCE) contains alkaloids, celluloses, flavonoids, lignins, triterpenoids, as principal bio-actives molecules, which can be regarded for their anticancer, antibacterial, and antioxidant activities [14,15,16,17,18]. The bio-active compounds present in *T. catappa* leaf extract (TCE) were reported as an excellent recipe for TCE mediated bio-reduction of Au^4+^, Cu^2+^, Ti^4+^, and Nd^3+^ to Au, Cu TiO_2_, and Nd_2_O_3_ NPs, respectively, wherein the extract functions as an excellent reducing and stabilizing agent [19,20,21,22,23]. However, despite the above reported protocols employed for the nanoscale fabrication of various metallic salts, silver which is usually the first choice among metallic nanomaterial fabrication due to its well-established microbicidal property, is yet to be prepared at nanoscale using *T. catappa* leaf extract (TCE) [24].

The antimicrobial potential of silver nanoparticles (Ag-NPs) is being investigated and is widely regarded as one of the most promising nanoantibiotics. This is due to interactions between Ag^+^ and cysteine moieties rich domains of cell proteins, which promote the loss of membrane potential through K^+^ loss, disrupting cellular transport and respiratory systems, and ultimately leading to bacterial death [25]. Chemical, physical, and green methods can be used to produce AgNPs. The resultant nanoparticles have a variety of properties (surface chemistry, shape, and size) that affect their capacity to combat bacteria and fungi [26]. They represent a new research approach that focuses on a new class of materials with prospective applications in the biomedical, biological, and pharmaceutical fields, and they have shown considerable promise and implications in the treatment of bacterial infections. Combining AgNPs with modest amounts of antibiotics has been suggested to boost their antimicrobial efficacy, providing outstanding results in vitro, and so holding potential for efficient in vivo bacterial eradication [26]. These nanoparticles and their ions exert antimicrobial activity by causing damage to the biofilm structure and components, as well as impairing bacterial metabolism via a variety of mechanisms [27]. These nanoparticles approach the biofilm, penetrate it, migrate internally, and interact with key biofilm components such as polysaccharides, lipids, proteins, and nucleic acids via hydrogen-bonding, electrostatic, ionic hydrophobic, Van der Waals interactions, while exerting antimicrobial activity [27]. AgNPs based on cyclophanes have recently been employed to recognize a wide range of biomolecules in aqueous conditions, including hydroxyl acids, amino acids, proteins, and nucleic acids. Colorimetric and electrochemical sensors based on cyclophane-capped AgNPs were also developed for the selective measurement of heavy metal cations, anions, various amino acids, pesticides, and polycyclic aromatic hydrocarbons. Cyclophane-capped AgNPs have shown promise in medicine and diagnostics [28]. At low doses, Ag^+^ ions show excellent biocompatibility with mammalian cells and are therefore often regarded as environmentally friendly [29]. Besides, bacteria against free Ag^+^ exhibit weak ability to augment resistance, which in fact offers a broad scope to explore alternatives for advanced antimicrobial formulations development against the rapidly growing antimicrobial resistance (AMR) globally [30].

Hence, with the aforementioned potentials of AgNPs and TCE bio-actives, the objective of the present study was: (i) Biofabrication of stable TCE-AgNPs by employing one step bio-inspired method; (ii) characterization of biosynthesised TCE-AgNPs by UV-Vis spectrophotometer, Fourier transform infrared (FTIR), Raman spectroscopy, transmission electron microscope (TEM), and scanning electron microscope (SEM); (iii) Investigation of antimicrobial activities of TCE-Ag-NPs against Gram-negative multidrug resistant *Pseudomonas aeruginosa* (MDR-PA), Gram-positive methicillin resistant *Staphylococcus aureus* (MRSA), and yeast *Candida albicans* by broth dilution and well diffusion methods; (iv) Investigation of antibiofilm potential of TCE-Ag-NPs against MDR-PA, MRSA, and *C. albicans* by 96-well microtiter crystal violet assay; (v) Assessment of the effect of TCE-AgNPs on biofilm structure of tested strains by SEM; and (vi) Interaction and visualization of ultrastructural alteration caused by TCE-AgNPs in MDR-PA, MRSA, and *C. albicans* by SEM and TEM.

## 2. Materials and Methods

### 2.1. Preparation of T. catappa Leaf Extract (TCE)

The fresh leaves of *T. catappa* were washed with sterile de-ionized water and air-dried. The dried *T. catappa* leaves were ground into fine powder by using domestic mortar pastel. Then, 5 g of *T. catappa* powder was added to 100 mL of sterilized de-ionized water vortexed vigorously, and then boiled for 20 min on a heating plate. The cooled solution was filtered by Whatman No. 1 paper and stored at 4 °C for future use.

### 2.2. Bio-Synthesis of TCE-Ag-NPs

The synthesis of TCE-NPs was carried out by following the method detailed elsewhere [31]. Precisely, 1, 2, and 5 mL aqueous TCE were added to 9, 8, and 5 mL of silver nitrate AgNO_3_ (Sigma Aldrich, St. Louis, MO, USA), respectively, to achieve a final concentration of 0.01 M AgNO_3_ in 10 mL of each reaction mixer. At room temperature (25–30 °C), the three experimental solutions began to develop an increased gradient of brown color after 30, 25, and 15 min function of TCE concentration as compared to the pale yellow solution observed at zero time. The solutions were spun at 14,000 rpm for 10 min to obtain precipitated TCE-Ag-NPs. The pelleted TCE-Ag-NPs were washed with ethanol and dried at 60 °C to obtain powder TCE-Ag-NPs. The Ag NPs obtained from the reaction mixtures containing 1, 2, 5 mL of TCE were designated as TCE-Ag-1, TCE-Ag-2, and TCE-Ag-3, respectively. The TCE-Ag-NPs were stored in the dark to investigate the physico-chemical and antimicrobial properties.

### 2.3. Physico-Chemical Characterization of TCE-Ag-NPs

#### 2.3.1. UV-Visible Spectroscopy

The formation of TCE-Ag-1, TCE-Ag-2, and TCE-Ag-3 NPs in formulations was monitored by detecting characteristic SPR of nano silver in the range of 200–700 nm on a UV-Vis spectrophotometer (PG Instruments Ltd., Alma Park, Wibtoft, Leicestershire, UK).

#### 2.3.2. FTIR Spectroscopy of TCE-Ag-3

FT-IR (Cary 630 Agilent, Stevens Creek Blvd., Santa Clara, CA, USA) was employed to identify and compare the functional groups of bio-actives present in pristine TCE as well as adsorbed on the surface of TCE-Ag-3. In brief, dried *T. catappa* leaves powder and TCE-Ag-3 were mixed with KBr separately and the spectra were recorded in the range of 4000 and 400 cm^−1^ at room temperature with a resolution of 4 cm^−1^ [32].

#### 2.3.3. Raman Spectroscopy of TCE-Ag-3

The Raman spectrum of TCE-Ag-3 was collected by using Inspector Raman (DXR, Thermo Fisher Scientific, Madison, WI, USA) with a 785 nm laser diode, a 200–2000 cm^−1^ spectral range, and a 15 cm^−1^ resolution. The value of the most intense band in the spectrum of TCE-Ag-3 analyzed by processing crude data with the Sigma Plot version 11.2 (Systat Software, Inc., San Jose, CA, USA) data analysis software.

#### 2.3.4. Electron Microscopy-Based Analyses of TCE-Ag-NPs

The morphological properties of TCE-Ag-1, TCE-Ag-2, and TCE-Ag-3 nano formulations were explored by using a scanning electron microscope (SEM, JSM-6610LV, JEOL, Tokyo, Japan), at an accelerating voltage between 10 and 20 kV by following the protocol described elsewhere [8]. Besides, the morphology and size of TCE-Ag-1, TCE-Ag-2, and TCE-Ag-3 nano formulations were further explored based on transmission electron microscopy (TEM; Morgagni 268, FEI, Brno-Černovic, Czech Republic). The size of three TCE-Ag-NPs formulations was further analyzed by processing the TEM images with the Image J1 (University of Wisconsin, Madison, WI, USA) multidimensional image processing software.

### 2.4. Antibacterial Activities of TCE-Ag-NPs

#### 2.4.1. Assessment of Antibacterial Activities of TCE-Ag-NPs by Well Diffusion Assay

TCE-Ag-1, TCE-Ag-2, and TCE-Ag-3 NPs formulations were assessed for their antibacterial activity. Concisely, 100 µL of exponentially growing (1 × 10^7^ colony forming unit (CFU)/mL) MR-*S. aureus* (MRSA) and MDR-*P. aeruginosa* (MDR-PA) were spread on Luria-Bertani agar (LBA) plates. Whereas, *C. albicans* cells (5 × 10^6^ CFU/mL) were evenly spread on Sabouraud Dextrose agar (SDA) plates. The LBA and SDA layers in the plates were punched with a sterilized stub to create wells with a diameter of 6 mm. Soft agar (0.7%) was added to all the wells to seal the bottom leakage prior to adding nano formulations. A fixed volume i.e., 100 µL of TCE-Ag-1, TCE-Ag-2, and TCE-Ag-3 NPs formulations were added into the wells of MRSA, MDR-PA, and *C. albicans* plates separately. The zone of growth inhibition (ZOI) was determined by considering the diameter of zones after incubating the plates at 37 °C for 24 h.

#### 2.4.2. Determination of MIC, MBC and MFC Values of TCE-Ag-NPs against Test Bacterial and Fungal Strains

The determination of minimum inhibitory concentration (MIC), minimum bactericidal concentration (MBC), and minimum fungicidal concentration (MFC) values of TCE-Ag-1, TCE-Ag-2, and TCE-Ag-3 NPs formulations against MRSA, MDR-PA, and *C. albicans* strains were determined following the two-fold serial dilution procedure, described by Ansari et al. [33]. Briefly, the bacterial inoculums of ~1 × 10^7^ CFU/mL were treated with TCE-Ag-1, TCE-Ag-2, TCE-Ag-3 NPs in a concentration range of 3.90 to 500 μg/mL. In the case of *C. albicans*, MIC and MFC values were determined from 31.25 to 250 μg/mL of TCE-Ag-NPs. The treated and untreated control tubes with bacterial and *C. albicans* strains were incubated for 24 h at 37 and 28 °C, respectively. The MIC was defined as the lowest concentration of antimicrobial agents that yielded no visible growth of the microorganisms [33]. For MBC/MFC determination, 100 μL aliquots from the culture tubes in which no visible growth was observed were spread on the LBA and SDA plates. The plates were then incubated for 24 h at 37 and 28 °C, respectively. The MBC/MFC endpoint is defined as the lowest concentration of antimicrobial agent that kills 100% of the initial microbial population [10].

#### 2.4.3. SEM Based Imaging of Test Strains and TCE-Ag-3

Significant higher antimicrobial activity of TCE-Ag-3 as compared to TCE-Ag-2 and TCE-Ag-1 formulations prompted us to investigate the TCE-Ag-3 induced morphological damages in planktonic cells of test strains. Hence, SEM imaging was performed with the MIC values of TCE-Ag-3 for MRSA, MDR-PA, and *C. albicans* cells. The untreated control and treated cells of test strains were incubated with TCE-Ag-3 for 24 h at 37 °C. Thereafter, cells spun at 5000 rpm for 5 min and incubated in glutaraldehyde (2.5%) at 4 °C for 4 h. The fixed cells were then incubated in 30%, 50%, 70%, and 90% ethanol for 15 min, consecutively. Then, 100 μL of each sample was uniformly spread on cleaned glass covers separately, dried at room temperature, sputter coated with gold, and finally examined by SEM (JSM-6610LV, JEOL, Tokyo, Japan).

#### 2.4.4. TEM Based Assessment of Antimicrobial Activity of TCE-Ag-3

The results of the SEM were confirmed by TEM analysis, as discussed elsewhere [10]. Briefly, MRSA, MDR-PA, and *C. albicans* cells grown in the presence of MIC values of TCE-Ag-3 were centrifuged, washed with MQ water, fixed in 2.5% glutaraldehyde and 1% osmium tetraoxide, and embedded in Epon resin (Polybed 812). So prepared blocks of cells were sectioned, stained with uranyl acetate and lead citrate (Sigma Aldrich, St. Louis, MO), and imaged on TEM (Morgagni 268, FEI, Czech Republic) with accelerating voltage of 80 kV.

#### 2.4.5. Evaluation of Antibiofilm Activity of TCE-Ag-3 against Test Strains

The 96-well microtiter plate method was used to evaluate the antibiofilm activity of TCE-Ag-3 NPs against MRSA, MDR-PA, and *C. albicans* [10]. Precisely, the wells seeded with 100 μL of bacteria (~1 × 10^7^ CFU/mL) and *C. albicans* (~5 × 10^6^ CFU/mL) were treated with 0.95, 1.95, 3.90, and 7.8 µg/mL of TCE-Ag-3 at 37 °C for 24 h. The wells were washed with phosphate buffer saline (PBS) after taking out the NPs suspensions. Subsequently, 200 μL of crystal violet (CV, 0.25%) was added for 30 min. The wells were washed, air dried, and filled with 95% ethanol to solubilize the biofilm bound CV. The absorbance was read at 595 nm by using a microplate reader.

#### 2.4.6. Effect of TCE-Ag-3 on Biofilm Structure of Tested Strains

In parallel, the destruction of biofilms matrices was analyzed by SEM following the protocol described in Section 2.4.3. Briefly, the biofilms adhered on the glass covers after 24 h of the treatment of TCE-Ag-3 (7.8 µg/mL) were fixed in 2.5% glutaraldehyde at 4 °C and dehydrated with 30%, 50%, 70%, and 90% ethanol for 15 min, consecutively. The biofilms were coated with gold sputtering before capturing the SEM micrographs.

## 3. Results and Discussion

### 3.1. T. catappa Leaf Extract (TCE) Mediated Synthesis of Ag-NPs

**Figure 1:**
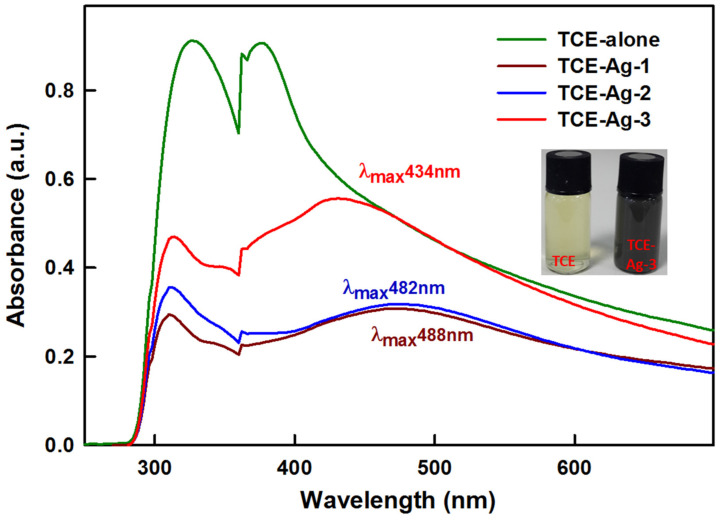
UV–vis analysis of synthesized TCE-Ag-NPs. The inset demonstrates the change in color of the reaction mixture from light yellow to dark brown nano Ag colloidal solution.

The role of bio-actives molecules contributed by animal, microbial, and plant cells and tissues in bio-inspired nanomaterial synthesis has been recognized, with the ultimate potential to reduce a wide range of metal cations to nano size particles [34,35,36]. Benign extracts of explants such as leaves, root, stem, bark, and seed typically act as bio-factory for the synthesis of a wide range of bio-actives including long and short hydrocarbon chains (saturated/unsaturated), aromatic complex cyclic compounds, polyphenols, alkaloids, flavonoids, terpenoids, sugars, proteins, and enzymes naturally [8,10,30,31]. Besides, easy availability, inexpensiveness, low toxicity, and indigenous therapeutic effects of plant bio-actives have attracted the nanotechnologies towards green synthesis approaches while developing their intended nanoconstructs. Albeit, *T. catappa* leaf extract (TCE) was investigated for TCE-capped Ag NPs. The inset in Figure 1 exhibited a steady change in color of aqueous TCE and AgNO_3_ (1:1) reaction mixture from pale yellow to dark brown after 15 min at 37 °C, which is regarded as a preliminary indication of TCE-Ag-NPs formation. This change in color was likely due to collective oscillation of conduction electrons of TCE-Ag-NPs produced in TCE-Ag-1, TCE-Ag-2, and TCE-Ag-3 reaction mixture, which darkens steadily with their age [37]. Compared to pristine TCE, the appearance of sharp peaks at 488, 482, and 434 nm in UV-vis spectra (Figure 1) can be assigned to SPR, developed by TCE bio-actives mediated reduction of Ag^+^ in reaction mixtures, respectively. At the same time, the formation of TCE-Ag-1, TCE-Ag-2, and TCE-Ag-3 can be regarded as a function of concentration of TCE. The increase in TCE concentration accelerated the reduction rate of Ag^+^ in reaction mixtures of TCE-Ag-1, TCE-Ag-2, and TCE-Ag-3, respectively. Furthermore, the isotropy in the nascent particle’s spherical shape and homogeneous distribution with no agglomeration was justified by a single SPR band in nano formulations, which can be attributed to efficient capping of TCE bio-actives around NPs.

### 3.2. FTIR Spectral Analysis of TCE-Ag-3

**Figure 2:**
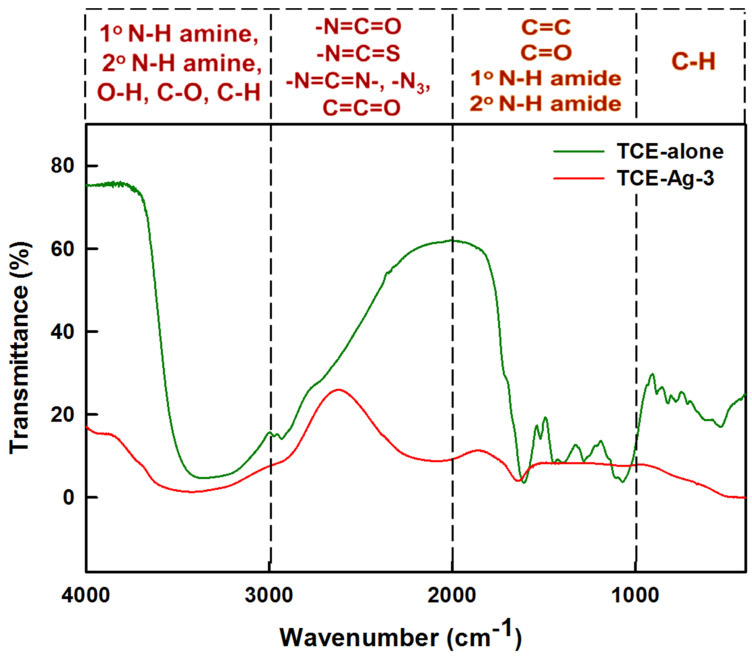
Comparison of FTIR spectra of TCE and TCE-capped Ag−3.

The FTIR spectra in Figure 2 demonstrated the characteristic vibrational/bending signatures of functional groups associated with bio-active moieties in the pristine TCE as well as adsorbed on Ag-NPs surface. The comparison of TCE and TCE-Ag-3 FTIR spectra indicates the involvement and potential role of bioactive molecules of TCE in AgNPs synthesis, capping, and stability. Strictly, the appearance of sharp bands between 2800 and 3700 cm^−1^ is likely due to stretching of aromatic, aliphatic H-bonded -OH, and polymeric -OH stretching and C-O groups present in TCE corona around TCE-Ag-3 [38]. Besides, vibrational signals around 1634, 1250, 1110, and 675 cm^−1^ correspond to the involvement of amide-I associated C=O group, C-H, and O-H groups deformation and bending of C-H bonds, respectively. Overall, the FTIR spectral signatures reflects the role of bio-actives phenolic carboxylic and alkene TCE in Ag^+^ reduction and encapsulation of nascent TCE-Ag-3 [39,40,41].

### 3.3. Raman Spectral Analysis of TCE-Ag-3

**Figure 3:**
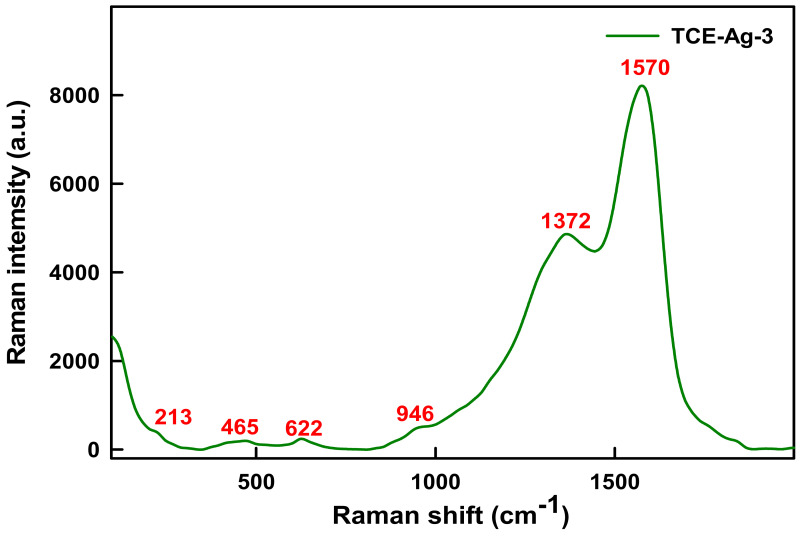
Raman spectroscopic analysis of TCE bio-actives plausibly capped of TCE-Ag-3.

In addition to FTIR, Raman spectroscopy was also used to examine the functional groups involved in the synthesis and stabilization of the as-prepared TCE-Ag-3 NPs. In fact, Raman spectroscopy relies on quenching of fluorescent bio-actives molecules by using metallic NPs to amplify the Raman signal [42]. Interestingly, earlier Raman spectroscopy reports suggest that -OH group positioned at 3 in flavonoids is most reactive in the presence of Ag-NPs and prompts native flavonoids to change their chemical structure readily [43]. The spectrum obtained from TCE-Ag-3 with the 785 nm laser was very similar to those obtained by Garcia-Bucio et al. [44]. The bands at 1570, 1372, 946, 622, and 465 cm^−1^ in Figure 3 are pretty similar to those that have been reported for flavones like morin (C_15_H_10_O_7_) corresponding to the bands at 1570 and 1372 cm^−1^, luteolin (C_15_H_10_O_6_) corresponding to 946 and 465 cm^−1^, and chrysin (C_15_H_10_O_4_) corresponding to 622 cm^−1^ in extract of *Cuscuta tinctoria* and *T. catappa* [43,44,45]. Jones et al. [46] have regarded certain bio-actives molecules viz quercetin, luteolin, phloretin, and morin as health beneficial flavonoids found in commonly consumed plant-based products. Morin, in the extracts of almond whole seed, brown skin, and green shell cover extracts, has been reported as a major flavonoid [47]. Harnly et al. [48] also reported the flavone, namely luteolin, in a significant amount in the extract of nuts and almond. Chrysin, a flavone, has not been reported before in almond extract, however, it is present in some natural products such as sunflower honey, propolis [49]. However, functional group bond behaviors present a parallel scenario, as summarized in Table 1. The two prominent broad peaks at 1570 and 1372 cm^–1^ correspond to C=C and C=O stretching vibrations at ring B and A rings of morin (C_15_H_10_O_7_) [41], whereas feeble signals at 946 and 465 cm^−1^ correspond to cyclic CH and COH bending in luteolin (C_15_H_10_O_6_) structure [50]. The signals at 622 cm^–1^ correspond to δ (CCO) and δ (COC) vibrations in chrysin (C_15_H_10_O_4_) rings [44]. Whereas, the weak signal at 213 cm^−1^can be assigned to the Ag–N and Ag–O bonds stretching [50,51].

### 3.4. Morphological Characterization of TCE-Ag-NPs

**Figure 4:**
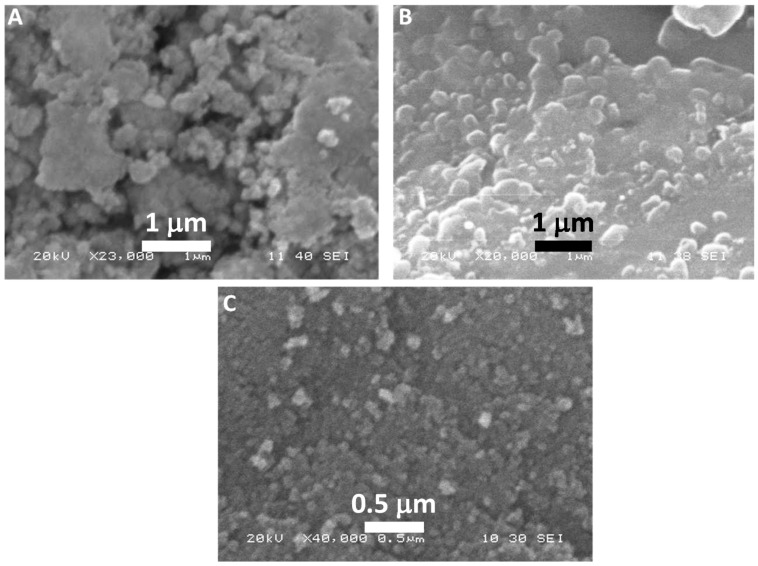
SEM-based comparative analysis of morphologies of TCE-Ag-1 (**A**), TCE-Ag-2 (**B**), and TCE-Ag-3 (**C**) NPs.

As prepared, colloidal formulations of TCE-Ag-1, TCE-Ag-2, and TCE-Ag-3 were analyzed by SEM and TEM in order to assess the shape and size of NPs. The SEM micrographs in Figure 4 demonstrate that the TEC-Ag-NPs were in pleomorphic shapes with a predominant number of spherical particles. Precisely, SEM micrograph of TCE-Ag-1 (Figure 4A) revealed the significant large size and irregular shapes of NPs, which is likely due to steady agglomeration after nucleation in the presence of least concentration of TCE (1 mL) bio-actives. However, as the ratio of capping bio-actives increased, i.e., 2 and 5 mL in the reaction mixtures of TCE-Ag-3 and TCE-Ag-3, the time of reaction and size of NPs were found to have reduced significantly, respectively. Besides, the SEM results presented in Figure 4B,C also revealed the presence of well diversed and spherical shaped NPs in TCE-Ag-2 and TCE-Ag-3 formulations, respectively. This increased dispersity of NPs in colloidal solutions can be argued as a result of significant capping of TCE bio-actives around nascent NPs which may have controlled the particle’s growth and agglomeration. Recently, Ranoszek-Soliwoda et al. [52] also demonstrated a similar pattern in size and agglomeration reduction in Ag-NPs with an increase in sodium citrate concentrations.

**Figure 5:**
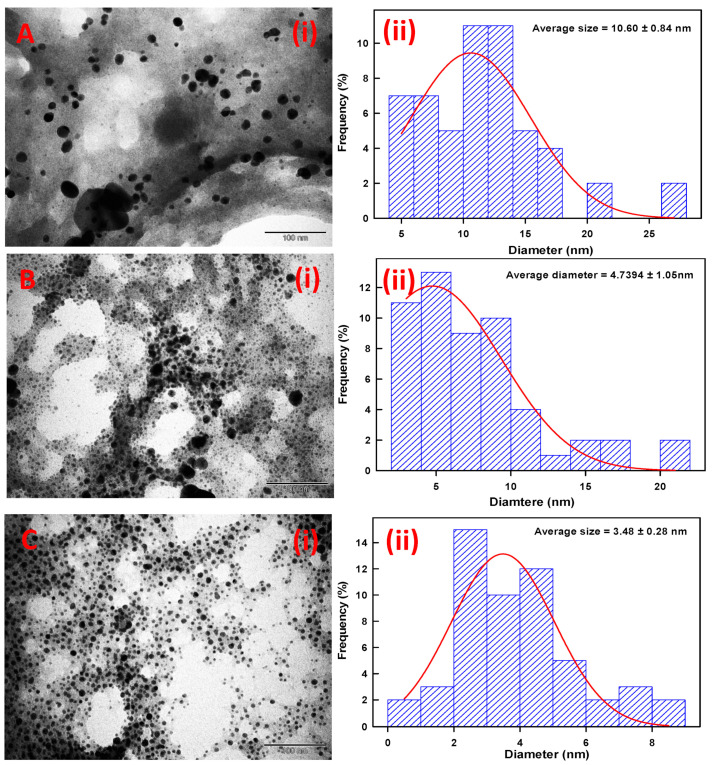
TEM and ImageJ-based comparative analysis of TCE-Ag-NPs. Panels (**i**) in (**A**–**C**) demonstrate TEM images of TCE-Ag-1, TCE-Ag-2, and TCE-Ag-3 NPs, respectively. Whereas, the histograms shown in panels (**ii**) in (**A**–**C**) demonstrate the average particle size and particle size distribution in the corresponding TEM images, respectively.

Similarly, TEM micrographs of TCE-Ag-1, TCE-Ag-2, and TCE-Ag-3 were also found in good agreement with SEM results. Compared to TCE-Ag-1 (Figure 5(Ai)), TEM images of TCE-Ag-2 (Figure 5(Bi)) and TCE-Ag-3 (Figure 5(Ci)) revealed a clear-cut reduction in size and agglomeration while maintaining their spherical shape. Nascent Ag-NPs formed at an early age of the reaction ultimately act as building-block of large NPs while growth proceeds. Whereas, considering the reaction phases (viz reduction, nucleation, growth, and stabilization), a point of time can be speculated during reduction where complex of Ag^+^-TCE bio-actives and free Ag^+^ ions exist. However, an increase in TCE bio-actives is expected to reduce unspent or free Ag^+^ while elevating the reduction and nucleation of reduced Ag^0^ into NPs due to readily available free TCE bio-active moieties for capping. Overall, it can be argued as we increased the TCE concentration, the (i) reduction of Ag^+^ to Ag^0^ and (ii) nucleation of Ag^0^ into NPs increased, whereas (iii) the growth of particles was controlled due to capping of TCE bio-actives around nascent NPs. Software (ImageJ)-based analysis of TEM images of TCE-Ag-1, TCE-Ag-2, and TCE-Ag-3 has also confirmed the TCE concentration dependent size control. Parallel ImageJ results demonstrate the average size of NPs as 10.06 ± 0.84 nm, 4.73 ± 1.05 nm, and 3.48 ± 0.28 nm for TCE-Ag-1 (Figure 5(Aii)), TCE-Ag-2 (Figure 5(Bii)), and TCE-Ag-3 (Figure 5(Cii)), respectively.

### 3.5. Antimicrobial Activities

#### 3.5.1. Assessment of Size-Specific Antimicrobial Activities by Well Diffusion Assay

**Figure 6:**
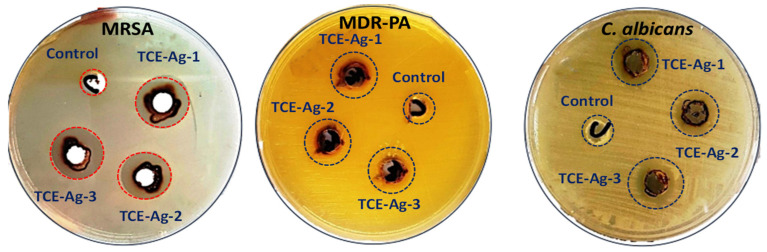
Comparative assessment of antimicrobial activity of TCE-Ag-1, TCE-Ag-2, and TCE-Ag-3 NPs against Gram-negative multidrug resistant *P. aeruginosa* (MDR-PA), Gram-positive methicillin resistant *S. aureus* (MRSA) and *C. albicans* cells.

The antimicrobial activity of TCE-Ag-1, TCE-Ag-2, and TCE-Ag-3 were tested against MR-*S. aureus* (MRSA) and MDR-*P. aeruginosa* (MDR-PA) and *C. albicans*. The images in Figure 6 exhibit the zone of growth inhibition produced with the 100 µL of crude formulations of TCE-Ag-1, TCE-Ag-2, and TCE-Ag-3 against test strains. The data summarized in Table 2 suggested that treatment of 100 µL of TCE-Ag-1 (10.60 nm) formulation inhibited the growth of MRSA, MDR-PA, and *C. albicans* as 11.07 ± 0.25, 19.03 ± 0.21, and 14.17 ± 0.06 mm, respectively. Similarly, the diameters of growth inhibition zones with TCE-Ag-2 (4.73 nm) formulation were found to be 12.13 ± 0.35, 20.20 ± 0.20, and 15.03 ± 0.15 mm against MRSA, MDR-PA, and *C. albicans*, respectively. The experiments with TCE-Ag-3 (3.48 nm) formulation yielded 14.23 ± 0.15, 21.13 ± 0.15, and 17.03 ± 0.21 mm diameters against MRSA, MDR-PA, and *C. albicans*, respectively. Overall, the zones of growth inhibition reflect the size dependent antimicrobial activity of TCE-Ag NPs against both bacterial as well as fungal strains. Our findings on the size and dose dependent antibacterial activity of TCE-Ag NPs are consistent with previously published research [53,54]. According to the recent work by [10,55,56], AgNPs synthesized by various methods have also antimicrobial action against Gram-negative and Gram-positive bacteria, as well as yeast, *C. albicans*. Precisely, as the size of TCE-Ag NPs decreased to 10.60 nm (TCE-Ag-1), >4.73 nm (TCE-Ag-2), and 3.48 nm (TCE-Ag-3), the diameter of growth inhibition increased 11.07 ± 0.25 mm, <12.13 ± 0.35 mm, and <14.23 ± 0.15 mm; 19.03 ± 0.21 mm, <20.20 ± 0.20 mm, and <21.13 ± 0.15 mm; and 14.17 ± 0.06 mm, <15.03 ± 0.15 mm, and <17.03 ± 0.21 mm, against MRSA, MDR-PA, and *C. albicans*, respectively (Table. 2). However, within bacteria, Gram-negative MDR-PA was found more sensitive against all three TCE-AgNPs formulations as compared to Gram-positive MRSA under identical conditions. However, there are no universal conclusions accepted to address the toxicity mechanism of Ag-NPs against microorganisms [57]. A Significant number of studies have claimed that Ag^+^ released after treatment dictates Ag-NPs toxicity [58,59]. Contrarily, it has also been debated that released Ag^+^ is not sufficient to augment toxicity induced by Ag-NPs whereas, the size, shape, surface coating, and surface charge can play an important role in influencing the extent of antimicrobial properties of nano structures [60,61,62].

#### 3.5.2. Size-Specific MIC, MBC, and MFC Values Determination of TCE-Ag-NPs against Test Strains

**Figure 7:**
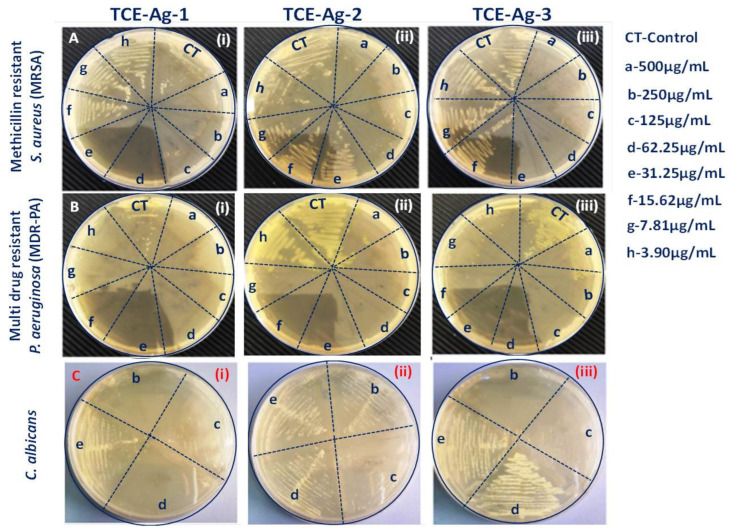
Comparative determination of MIC, MBC, and MFC values of TCE-Ag-1, TCE-Ag-2, and TCE-Ag-3 NPs against MDR-PA, MRSA, and *C. albicans* cells.

The data in Table 3 described the size dependent MIC and MBC values of TCE-Ag-1 (10.60 nm), TCE-Ag-2 (4.73 nm), and TCE-Ag-3 (3.48 nm) NPs for cultured MRSA, MDR-PA, and *C. albicans* cells. At an identical concentration i.e., 100 µL of TCE-Ag-1 (10.60 nm), >TCE-Ag-2 (4.73 nm), and >TCE-Ag-3 (3.48 nm) NPs, MRSA, MDR-PA, and *C. albicans* cells grown for 24 h demonstrate the decrease in their MIC and MBC or MFC values with the decrease in size of NPs. Briefly, the MIC and MBC values of TCE-AgNPs having an average diameter of 10.60, 4.73, and 3.48 nm against Gram-positive MRSA cells grown for 24 h were determined to be 31.08 ± 1.01 and 124.33 ± 4.04 µg/mL, 7.77 ± 0.25 and 31.08 ± 1.01 µg/mL, and 7.77 ± 0.25 and 31.08 ± 1.01 µg/mL, respectively (Table 3; Figure 7(Ai–Aiii)). Similarly, MIC and MBC values for Gram-negative MDR-PA were also affected by the decrease in size (10.60>, 4.73>, and 3.48 nm) of TCE-Ag-1, TCE-Ag-2, and TCE-Ag-3 as 7.77 ± 0.25 and 15.54 ± 0.50 µg/mL, 3.88 ± 0.13 and 7.77 ± 0.25 µg/mL, and 3.88 ± 0.13 and 7.77 ± 0.25 µg/mL, respectively (Table 3; Figure 7(Bi–Biii)). Compared to bacteria, the MIC and MFC values of TCE-Ag-1 (10.60 nm), TCE-Ag-2 (4.73 nm), and TCE-Ag-3 (3.48 nm) NPs for *C. albicans* were found significantly higher as 124.33 ± 4.04 and 248.67 ± 8.08 µg/mL, 62.17 ± 2.02 and 248.67 ± 8.08 µg/mL, 62.17 ± 2.02 and 124.33 ± 4.04 µg/mL, respectively (Table 3; Figure 7(Ci–Ciii)). The MICs and MBCs/MFCs values of AgNPs in this study are comparatively lower than those found in previous studies [10,56]. Alqahtani et al. [56] determined that biogenic AgNPs had MIC and MBC values of 39–78 g/mL and 156–312 g/mL, respectively, against MDR-*P. aeruginosa* and MRSA. In another work, Ansari et al. [10] reported that green synthesized AgNPs had that MIC and MBC/MFC values of 258.3 ± 14.4–758.3 ± 38.2 µg/mL and 516.7 ± 28.9–1533.3 ± 57.7 µg/mL against MDR-PA, MRSA, and *C. albicans*, respectively.

#### 3.5.3. SEM and TEM Based Analysis of the Interaction of TCE-Ag-3 with Planktonic Cells of Test Strains

**Figure 8:**
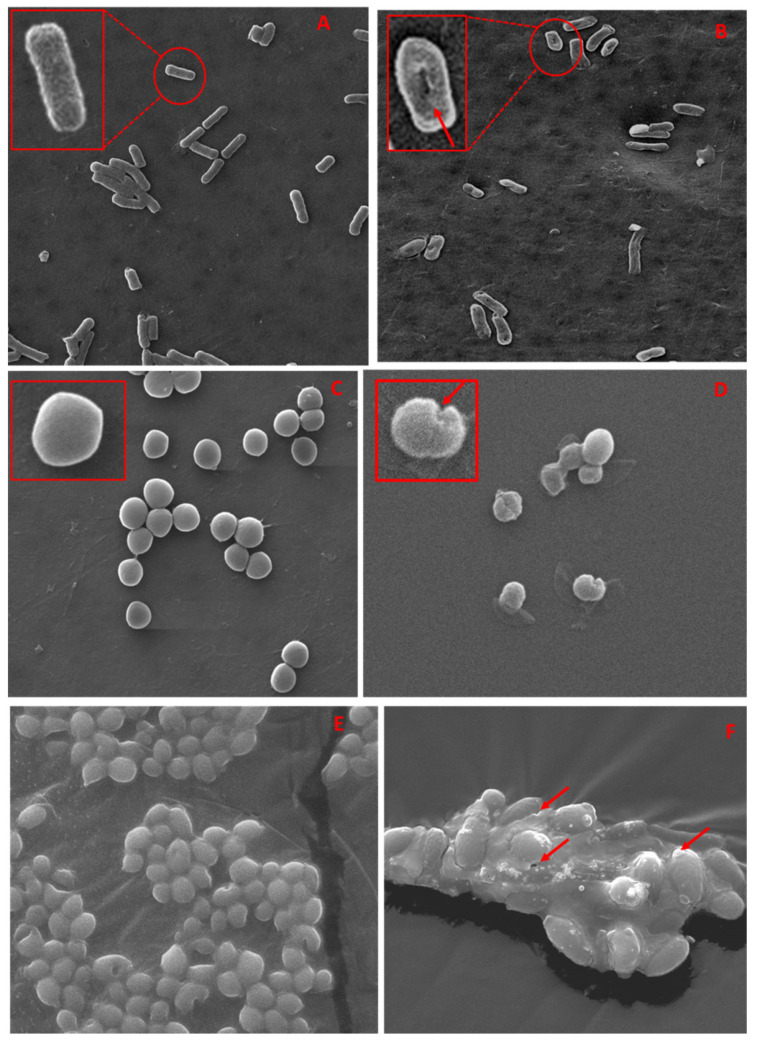
SEM micrographs showing morphological changes in Gram-negative multidrug resistant MDR-PA cells (**A**,**B**); Gram-positive methicillin resistant MRSA cells (**C**,**D**) and *C. albicans* cells (**E**,**F**), respectively, in the absence the (**A**,**C**,**E**) and presence (**B**,**D**,**F**) of TCE-Ag-3 NPs.

**Figure 9:**
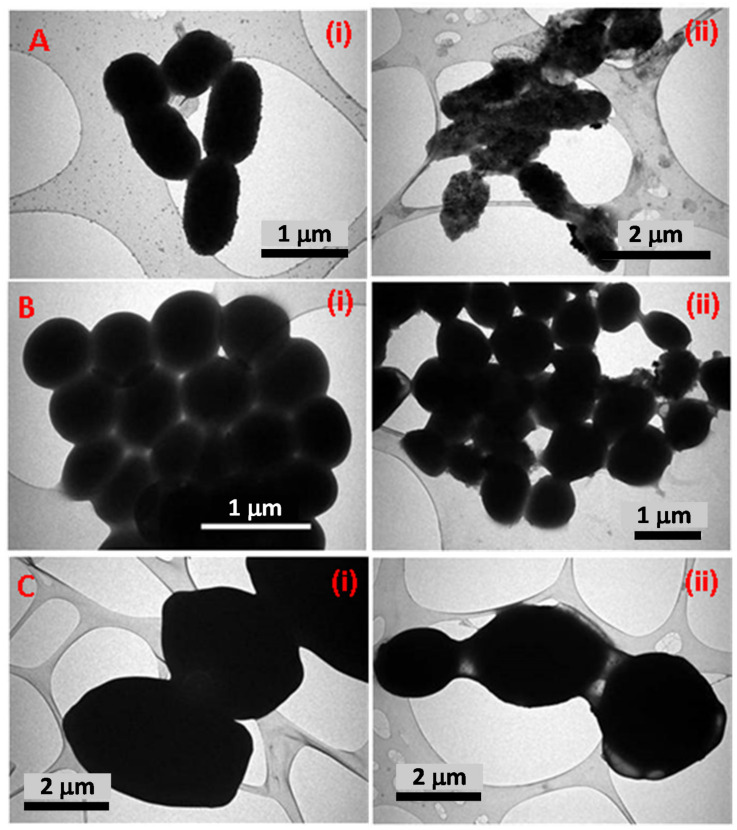
Shows a TEM-based comparative analysis of the interaction of TCE-Ag-3 NPs with test microbial cells. Panels (**i**) in (**A**–**C**) show TEM images of untreated MDR-PA, MRSA, and *C. albicans* cells, whereas panels (**ii**) in (**A**–**C**) show TEM images of MDR-PA, MRSA, and *C. albicans* cells treated with their MIC of TCE-Ag-3 NPs after 24 h.

To validate the obtained antimicrobial activity, we have also carried out a parallel assessment of interaction between TCE-Ag-3 NPs and test strains by SEM and TEM analyses. Owing to differences in the structure and thickness of the peptidoglycan layer between Gram-positive (~20–80 nm) and Gram-negative (~7–8 nm) bacteria, TCE-Ag-3 NPs exhibited significantly greater antibacterial effect against Gram-negative MDR-PA than Gram-positive MRSA. The representative SEM images demonstrate the TCE-Ag-3 NPs induced cellular damage as deep pits and cavities in Gram-negative MDR-PA cells (Figure 8B), whereas untreated control cells sustained their native rod shape and intact cell wall (Figure 8A). TCE-Ag-3 NPs treated Gram-positive MRSA cells in Figure 8D show significant cellular damage as compared to control cells with smooth and spherical shapes (Figure 8C). The clear-cut reduced cellular damage in MRSA cells is most likely due to its rigid and three-dimensional peptidoglycan layer which is composed of peptides mediated by cross-linked polysaccharide chains [63]. The exact antimicrobial mechanism of the action of AgNPs remains unclear. It has been suggested that AgNPs could interact with the cell wall, which could lead to pits or might go deeper, into the bacterial cell, where they could influence metabolic processes i.e., by interfering with oxidative phosphorylation or forming complexes with nucleic acids [64]. It has been suggested that modifying the surface charge of AgNPs could enhance their antibacterial activity [65]. It is well known that the bacterial cell membranes have a negative charge due to the presence of various negative charge molecules such as carboxyl, amino, hydroxyl, and phosphate groups [66]. The negatively charged molecules in the cell membrane promote AgNP adhesion, making bacteria more vulnerable to antimicrobial therapy [29]. Abbaszadegan et al. [66] reported that positively charged AgNPs were attracted to negatively charged bacterial cell membranes because of electrostatic interactions, causing the positively charged nanoparticles to be adhered to the bacteria’s cell wall and membranes. [66]. Furthermore, in another study, AgNPs with a negatively charged surface have been investigated and shown to be more effective in inhibiting the growth of *S. aureus* [64]. Therefore, it has been proposed that when these types of interactions occur, morphological changes such as cytoplasm shrinkage and membrane detachment occur, which ultimately leads to cell wall rupture and cell death as illustrated in Figure 8 and Figure 9. Similarly, we have also evaluated the antifungal potential of TCE-Ag-3 NPs against *C. albicans* cells by SEM. After 24 h of exposure of TCE-Ag-3 NPs, the *C. albicans* cells showed notable alterations such as loss of native size and shapes likely due to internalization of NPs as compared to control cells which exhibited intact cell membrane and the cell wall (Figure 8E,F). The cytoplasmic volume of treated cells increased significantly with a remarkable cell membrane invagination (Figure 8F). Similarly, the TEM based analysis of TCE-Ag-3 treated MDR-PA, MRSA, and *C. albicans* cells have also shown significant incorporation of NPs on the surface and cellular damage as compared to their untreated cells (Figure 9(Aii–Cii)).

#### 3.5.4. Antibiofilm Activity of TCE-Ag-3 against Test Strains

Colonization of planktonic microbial cells of bacteria and fungi eventually develops into multispecies biofilms causing the failure of a wide range of antibiotic efficacy. Precisely, harsh micro-environments of biofilms generally demonstrate a complex set of favorable conditions for prolonged biofilm survival while protecting from antimicrobial agents likely due to mechanical support of thick EPS layer around persistent microbial cells. Besides, enzymatic and acidic degradation of various antimicrobial agents has also been identified as a notorious mechanism of defence [6]. Thus, this delayed or compromised approach of microbial agents to biofilm persistent led them to develop MDR [67,68]. However, under said biofilm environments, a broad range of metallic NPs have been regarded as a promising alternative strategy to circumvent chronic biofilm infections. Concisely, the enhanced penetrability, high payload of drugs, stability, and post-dissolution release of metal cations in the acidic and enzymatic vicinities of biofilm matrices have generally been credited for promising antimicrobial activity of metallic nano constructs. Nevertheless, we have evaluated the antibiofilm potential of as-prepared TCE-Ag-3 nano formulations against test microorganisms in this study.

**Figure 10:**
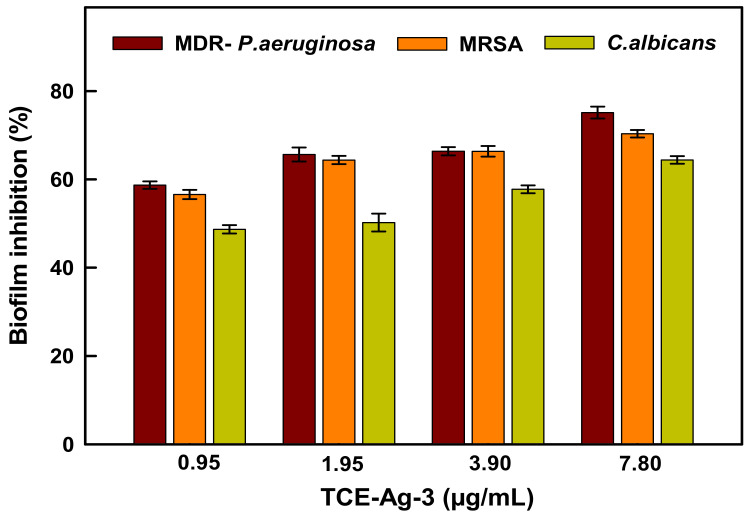
Reduction in the formation of biofilm in the presence of 0.95, 1.95, 3.90, and 7.80 μg/mL concentrations of TCE-Ag-3 NPs. The error bars represent standard deviations of triplicate samples.

**Figure 11:**
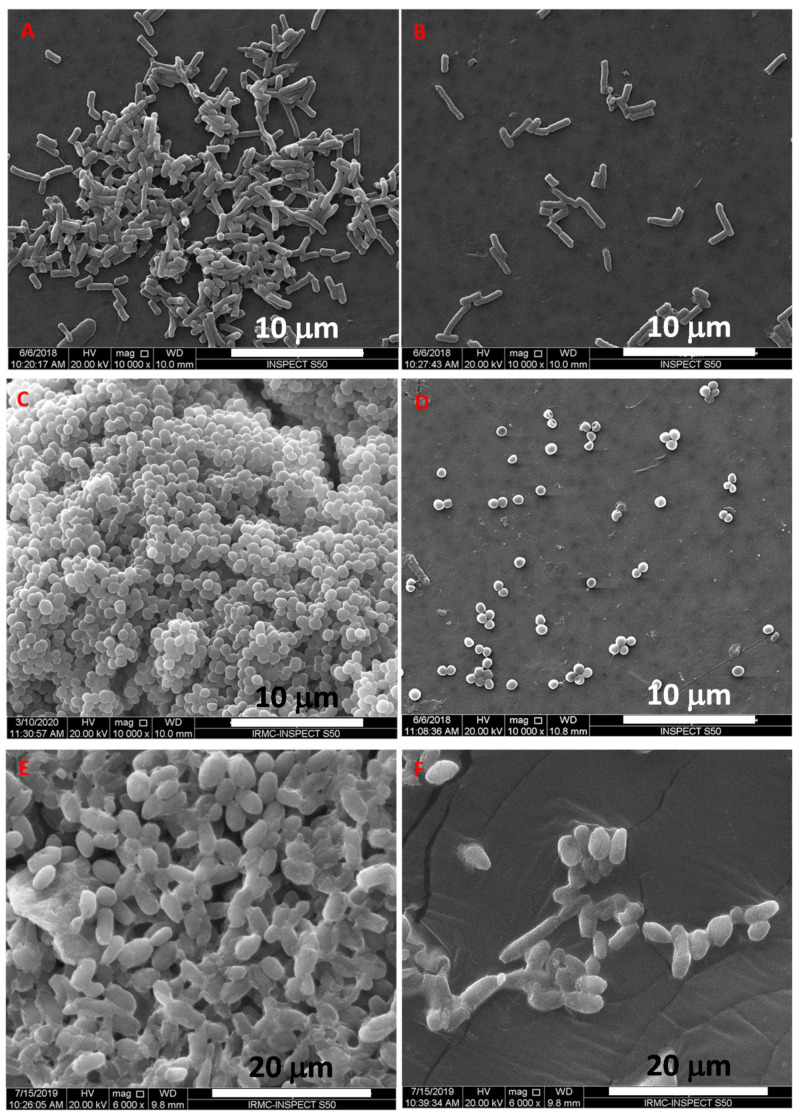
SEM images showed significant depletion in MDR-PA (**B**), MRSA (**D**), and *C. albicans* (**F**) biofilm formation, respectively, at 7.80 μg/mL of TCE-Ag-3 NPs after 24 h as compared to untreated experiment (**A**,**C**,**E**).

The bar graph in Figure 10 demonstrates the TCE-Ag-3 dose-dependent inhibition of biofilm formation. The data showed 58.70 ± 0.8%, 65.6 ± 1.5%, 66.4 ± 0.9%, and 75.14 ± 1.3% reduction of biofilms formed by MDR-PA as compared to untreated control (100%), at 0.95, 1.95, 3.90, and 7.80 µg/mL after 24 h of treatment, respectively. Under identical conditions, 56.58 ± 1.05%, 64.39 ± 0.95%, 66.38 ± 1.19%, and 70.35 ± 0.85%; and 48.70 ± 0.94%, 50.23 ± 2.0%, 57.76 ± 0.89%, and 64.41 ± 0.87% inhibition was observed in MRSA and *C. albicans* biofilms formation, respectively (Figure 10). Both, the bacterial and fungal strains are known to be notorious biofilm producers, which complicates their management in clinical and healthcare settings. Whereas, the parallel SEM analysis carried out at 7.8 µg/mL of TCE-Ag-3 has demonstrated a notable destruction in biofilms of MDR-PA (Figure 11B), MRSA (Figure 11D), and *C. albicans* (Figure 11F) as compared to matured biofilms in their control experiments (Figure 11A,C,E). They are well-known causative agents for several health concerns in humans, beginning with mild superficial skin infections to death leading to visceral or bone infections [69]. The rapid increase in methicillin-resistance and failure of available antibiotics has made such chronic infections more challenging against conventional treatment modalities. Hence, the biofilm inhibition in TCE-Ag-3 treated experiments prompt to envision the TCE-Ag-3 NPs as a viable option for controlling biofilm related microbial infections. Indeed, the bio-actives tannins in TCE have been regarded as a potential anti-quorum sensing (QS) and biofilm controlling plant organic agent against *Chromobacterium violaceum* and *P. aeruginosa* [70]. Besides, *C. albicans*, a naturally occurring pathogen in the oral cavity, was found to attenuated significantly (*p* < 0.001) while causing biofilm maturation on the denture acrylic surface at 6.25 mg/mL of TCE [71]. Although, it can be conceived that there are polyphenolic TCE bio-actives which have plausibly aggravated the QS-controlled biofilm maturation against microbial biofilms.

## 4. Conclusions

The findings show that the interaction of TCE bio-actives Ag^+^ resulted in the formation of TCE-Ag-1, TCE-Ag-2, and TCE-Ag-3 nano formulations through a simple, fast, cost-effective, and environmentally friendly path. The advantage of green synthesis of AgNPs based on plant extracts is that it contributes to environmental and human health protection by reducing the use of harmful and hazardous chemicals. The spectroscopic based art-of-the-techniques including UV-vis, FTIR, and Raman spectroscopy presented a three-tier confirmation of the involvement of TCE bio-actives in (i) Ag^+^ reduction into Ag-NPs, (ii) controlling the particle’s size, and (iii) stabilization of nascent particles by forming soft corona around NPs. In this study, the plant-derived TCE-AgNPs showed strong broad-spectrum antimicrobial and antibiofilm activity against different clinically important human pathogens such as MDR-PA, MRSA, and *C. albicans* at lower concentrations, suggesting that they could be used to treat infections caused by these biofilm-forming drug resistant organisms. Current antibiotics, as well as the antibiotic idea of combating pathogens, are increasingly becoming ineffective against new strains of existing pathogens and disease-causing species. High-throughput nanotechnology curated antimicrobials offer a novel approach to treating microbial pathogens and biofilms that are resistant to existing treatments. Because of the increasing number of AgNPs applications, more research is required to understand the toxicity and fate of AgNPs in ecosytems, as well as the related toxicity and safety issues, in order to ensure the safe use of AgNPs.

## Figures and Tables

**Table 1:** Band positions from TCE-Ag-3 colloidal solution at 785 nm laser wavelength.

Sample	Band Wave number/cm^−1^	Plausible Bond Assignments	Plausible TCE Bio-Active Molecules on NPs Surface	References
**TCE-Ag-3**	1570	C=C of ring B vibrations	Morin (C_15_H_10_O_7_)	[43,44,47]
	1372	C=O of ring A vibrations	Morin (C_15_H_10_O_7_)	[43,44,47]
	946	cyclic ring bend, cyclic CH, COH bend	Luteolin (C_15_H_10_O_6_)	[44,45,48,50]
	622	δ (CCO) and δ (COC) vibrations	Chrysin (C_15_H_10_O_4_)	[44]
	465	Cyclic CH, COH bend, CO(H) Ag bend, and CO_2_ twist	Luteolin (C_15_H_10_O_6_)	[44,45,48]
	213	Ag–O vibrations	Unassigned	[50]

**Table 2:** Antimicrobial activity of TCE-Ag NPs against test strains of bacteria and fungi.

Sr. No.	Bacterial Strains	Zone of Inhibition (mm)		
		TCE-Ag-1	TCE-Ag-2	TCE-Ag-3
1	MR-*S. aureus*	11.07 ± 0.25	12.13 ± 0.35	14 ± 0.15
2	MDR-*P. aeruginosa*	19.03 ± 0.21	20.20 ± 0.20	21.13 ± 0.15
3	*C. albicans*	14.17 ± 0.06	15.03 ± 0.15	17.03 ± 0.21

**Table 3:** MIC and MBC/MFC * (µg/mL) values of TCE-AgNPs against test strains of bacteria and fungi (* indicate MFC for *C. albicans*).

Strains	TCE-Ag-1		TCE-Ag-2		TCE-Ag-3	
	MIC	MBC/MFC *	MIC	MBC/MFC *	MIC	MBC/MFC *
**MRSA**	31.08 ± 1.01	124.33 ± 4.04	7.77 ± 0.25	31.08 ± 1.01	7.77 ± 0.25	31.08 ± 1.01
**MDR-*P. aeruginosa***	7.77 ± 0.25	15.54 ± 0.50	3.88 ± 0.13	7.77 ± 0.25	3.88 ± 0.13	7.77 ± 0.25
*C. albicans* *	124.33 ± 4.04	248.67 ± 8.08 *	62.17 ± 2.02	248.67 ± 8.08 *	62.17 ± 2.02	124.33 ± 4.04 *

## Data Availability

The data presented in this study are available in this manuscript.
